# Antimicrobial Usage and -Resistance in Livestock: Where Should We Focus?

**DOI:** 10.3389/fvets.2017.00148

**Published:** 2017-09-15

**Authors:** Ioannis Magouras, Luís P. Carmo, Katharina D. C. Stärk, Gertraud Schüpbach-Regula

**Affiliations:** ^1^Veterinary Public Health Institute, Vetsuisse, University of Bern, Bern, Switzerland; ^2^SAFOSO AG, Bern, Switzerland

**Keywords:** antimicrobials, resistance, livestock, public health, One-Health

Antimicrobials represent one of humanity’s medical revolutions enabling us to treat both human and veterinary bacterial infections. It is, therefore, of utmost importance to preserve their effectiveness. However, during the last decades, the continuing rapid development of antimicrobial resistance (AMR) has emerged as a major global public health concern (1). Resistant bacteria may hamper the treatment of infections resulting in prolonged illness, disability, and death (2).

In veterinary medicine, antimicrobials play a crucial role in the maintenance of animal health, animal welfare, and food-safety (3). However, a not yet quantifiable share of the burden of resistance for public health is attributable to the use of antimicrobials in livestock production (4–6). Farm animals are exposed to considerable quantities of antimicrobials (7) and can act as an important reservoir of AMR genes, which could be transmitted to humans through the food chain, direct animal contact and the environment. Use of antimicrobials in agriculture also includes those defined by the World Health Organization (WHO) as “critically important” for human medicine (8). Resistance against these substances can limit dramatically the treatment options against serious human bacterial diseases. Notorious examples include the vancomycin-resistant enterococci (VRE), the extended-spectrum β-lactamase (ESBL) producing *Enterobacteriaceae* and the recently detected plasmid-mediated colistin resistance (mcr-1 gene) in livestock, food, and humans in China (9–11).

Resistant bacteria can be introduced into the environment through several ways, such as the land application of livestock manure as fertilizer (12). The globally rising aquaculture sector, which is characterized by extensive use of antimicrobials, represents another important source of resistant bacteria that can find their way into the environment (13). Our understanding on the epidemiology of AMR in livestock production is also hampered by the lack of comprehensive antimicrobial usage (AMU) data in the majority of countries. Furthermore, AMR development and spread is driven by human behavior, from the prescription of antimicrobials to infection prevention and control. Understanding these factors is a major step toward fighting against AMR.

The complex epidemiology of AMR emphasizes the need for highly interdisciplinary research approaches, comprising humans, animals, and the wider environment. In line with the WHO global action plan on AMR (14), it is the authors’ opinion that research should be prioritized toward (a) understanding the social/behavioral drivers of AMU and AMR, (b) establishing or improving systems to monitor AMU, and (c) encouraging a holistic approach through the One-Health concept when addressing the phenomenon and risk of AMR.

## Social Sciences

It is well established that resistance to a new antimicrobial substance begins shortly after its introduction; therefore, development of new antimicrobials should not be viewed as the only solution to combat AMR (15). The emergence and spread of AMR is largely influenced by human behavior, which in turn is shaped by cultural, social, political, and economic factors (16). This is also evident in the wide variation across the globe in patterns of use and resistance to antimicrobials, which cannot always be explained by differences in the diseases present, in health care infrastructure or farming systems (17, 18). Therefore, social sciences can shed light on the multi-faceted reasons that lead to the application of antimicrobials and the development of AMR. Social sciences are also valuable in identifying the most impactful and feasible interventions to counteract the AMR phenomenon.

In livestock production, veterinarians and farmers play a preponderant role when it comes to AMU and AMR. In many cases, veterinarians decide whether to treat an animal or not with antimicrobials, select the antimicrobial to be used, as well as define the dosage and route of administration. Veterinarians also advise farmers on animal health, biosecurity and production management issues that can strongly influence animal health, AMU, and the transmission of resistant bacteria. Farmers are a source of valuable information on farm management, biosecurity, animal health, and welfare that could be used to identify risk factors (and consequently interventions) associated with AMU in livestock.

Surveys and expert opinions are well-accepted approaches for exploring the behavioral basis of AMU and AMR. These methods could provide informative data on the attitudes, motivation, and knowledge of veterinarians and farmers toward AMU and AMR (19). On the other hand, controlled experimental studies that assess the success of specific interventions are rarely conducted. This research area should be expanded to lay the foundations for the design and implementation of intervention strategies toward the reduction of AMU and AMR.

## Monitoring of AMU

Bacteria can be naturally resistant against specific antimicrobial classes (intrinsic resistance) (20), however in the majority of the times, it is the exposure to antimicrobials that provide the necessary selective pressure for the emergence and spread of resistant bacteria. It should be emphasized that non-antimicrobial agents, namely metals and biocides are also implicated in co-selection of AMR (21). Data collection on AMU is an indispensable step in our attempt to understand and fight AMR. Monitoring of AMU allows the analysis of temporal trends in antimicrobial consumption and can ensure compliance with prudent usage practices, programs, or regulations. Furthermore, monitoring systems can assist in identifying the most efficient interventions for optimizing AMU. In combination with AMR data, quantification of AMU can be useful not only in detecting risk factors for the emergence of resistance, but also in describing temporal associations between AMU and AMR. This would provide evidence on the link between AMU and AMR to researchers, as well as policy and decision makers. In addition, analyzing these data can provide a basis for targeted research and development. The need for standardized usage data of high quality and resolution has been stressed by the European Surveillance of Veterinary Antimicrobial Consumption (ESVAC) (22, 23). The abovementioned benefits of monitoring antimicrobial consumption can be boosted when data on consumption per species are available. However, the resource-demanding nature of such monitoring systems often combined with political and confidentiality issues explains why only a few countries, such as The Netherlands and Denmark, have nation-wide automated monitoring systems in place (24, 25). Monitoring systems that are based on the collection of farm level data allow for the implementation of benchmarking strategies. These make it possible to rank individuals (farmers or veterinarians) by their level of AMU and to implement measures in order to reduce consumption by the top users. This is an additional benefit, given that benchmarking strategies have been quite successful in reducing AMU in the countries that adopted them. Denmark and The Netherlands are among the countries which experienced a drop in antimicrobial consumption following the implementation of benchmarking systems (26, 27). Alternatives to automated systems include performing randomized field studies or extrapolating species’ consumption through sales data stratification (28). Nonetheless, automated collection of prescription/usage data should be preferred as long term goals.

## The Ecology of AMR and the Need for a One-Health Approach

The complex epidemiology of AMR together with the socio-economical drivers make this topic the quintessential One-Health issue. Transectoral and transdisciplinary approaches are a “must-do” to tackle AMR appropriately. A reduction in AMU was not always followed by a decline in AMR, as demonstrated in the case of VRE (6). Reducing the dissemination and transmission of resistant bacteria within and between animal and human populations is central when aiming to fight AMR. The ability of bacteria to disseminate from one setting to another, sometimes over large geographic distances and among the different populations, makes it difficult to explain with certainty the origin of resistant bacteria strains. Therefore, the reservoirs and the transmission pathways of antimicrobial-resistant bacteria merit further investigation, ideally through a One-Health approach.

Livestock trade creates a complex, heterogeneous, contact network that shapes between-herd transmission of infectious diseases. Direct transmission of resistant bacteria is well documented for livestock-associated methicillin-resistant *Staphylococcus aureus* (LA-MRSA). Here, animal trade has been identified to be a major driver of LA-MRSA dissemination (29, 30). For other bacteria, such as *Enterobacteriaceae* and in particular *Escherichia (E.) coli*, fecal shedding represents the main route of dissemination, thus not only host, but also environmental reservoirs may exist which constitute multiple, complex ways of resistance introduction and transmission. So far, experimental studies have demonstrated animal-to-animal transmission of resistant *E. coli* under controlled conditions within confined compartments (31). However, potential factors that drive transmission, such as farm management and the farm environment, have not been studied thoroughly for bacteria such as *E. coli* or *Enterococci*. The practice of land application of livestock slurry and manure represents a major source for introduction of resistant bacteria into the environment (12, 21). Animals can as well excrete resistant bacteria directly in the environment through their feces while being on pastures (32). *E. coli* spends approximately half of its life cycle in the external environment and, therefore, anything contaminated with these potentially antimicrobial-resistant bacteria may constitute a reservoir for their dissemination (33).

Wild animals are usually not treated with antimicrobials; however, they can carry antimicrobial-resistant bacteria from the farm’s surrounding contaminated environments. Wild animal species that acquire resistant bacteria could constitute an additional reservoir of AMR in the environment and could function as vectors (and eventually as amplifiers) for dissemination to other species, including humans (34).

It is, therefore, important to improve our knowledge on how animal contacts and trade (direct transmission), farm management, and the wider farm environment (indirect transmission) drive the dissemination of AMR and to identify potential interventions to counteract this phenomenon. Farm management studies could include all those practices that potentially facilitate spread of resistant bacteria within and between farms and from farms to the environment, such as farm hygiene and biosecurity, animal waste management, structure (and construction material) of holdings as well as animal production intensity.

Holistic, One-Health approaches should always be backed with molecular epidemiological data, which can provide information about links between resistance genes observed in different samples, such as from animals of different origin. Resistance genes should be studied not only in animal samples but also in the wider farm environment, such as farmers, other livestock species, farm pets, wildlife, manure, and water. These ecological data can provide the molecular link to characterize reservoirs of resistant bacteria and could support studies on transmission pathways between animal populations but also from animals to humans and *vice versa*. Source attribution can be of help to shed light on the contribution of AMR originating from livestock to the public health resistance burden. Moreover, it can also be an important piece of evidence when developing targeted interventions against AMR. Genomic data might also provide some additional information on potential evolutionary processes in bacteria during transmission within the studied populations. Furthermore, molecular epidemiology data can shed some light on how much of the resistance reservoir is attributed to the spread of resistant bacteria or *de novo* emergence due to AMU selection pressure in the studied farms.

## Conclusion and Prospects

AMR is a complex phenomenon and is driven by biological processes and socio-economical factors. Understanding the attitude and knowledge of farmers and veterinarians toward AMU and AMR is a crucial step for the design of strategies to combat this public health threat. The lack of detailed AMU data impacts our ability to interpret surveillance data on AMR and to design efficient interventions. Therefore, monitoring systems to fill this knowledge gap should be prioritized. Finally, the ecology of AMR should be addressed with a holistic, One-Health approach combining expertise from different disciplines, such as veterinary clinicians, public health scientists, microbiologists, wildlife veterinarians, environmental scientists (ecologists), agricultural/forestry scientists, and epidemiologists.

## Author Contributions

IM wrote the manuscript. LPC provided valuable expertise on monitoring systems for antimicrobial usage and social sciences. KS provided valuable expertise on the topics of One-Health and social sciences. GS-R provided valuable expertise and feedback in all topics included in this opinion manuscript and assisted IM in the conceptualization of the manuscript. All the authors have read and approved the manuscript.

## Conflict of Interest Statement

The authors declare that the research was conducted in the absence of any commercial or financial relationships that could be construed as a potential conflict of interest.
